# Dihexyl (2-(Hydroxyamino)-2-Oxoethyl) Phosphonate as a Novel Collector for Flotation Separation of Scheelite and Quartz

**DOI:** 10.3390/molecules30173607

**Published:** 2025-09-03

**Authors:** Jingjing Xiao, Pan Xiao, Yongjun Miao, Sisi Liu, Jia Tu, Qing Tang, Changzhu Li, Zhihong Xiao, Rukuan Liu

**Affiliations:** 1 State Key Laboratory of Woody Oil Resources Utilization, Hunan Academy of Forestry, Changsha 410004, China; xjj0806@hnlky.cn (J.X.); yjmiao1971@126.com (Y.M.); liusisi274@126.com (S.L.); liurukuan@gmail.com (R.L.); 2 The College of Resources and the Environment, Central South University of Forestry and Technology, Changsha 410004, China; 19146808384@163.com; 3 Center for Industrial Analysis and Testing, Guangdong Academy of Sciences, Guangzhou 510645, China; tangqing822@163.com

**Keywords:** scheelite, quartz, dihexyl (2-(hydroxyamino)-2-oxoethyl) phosphonate, flotation selectivity

## Abstract

In this paper, a novel collector dihexyl (2-(hydroxyamino)-2-oxoethyl) phosphonate (DHHAOEP) was synthesized and used as a flotation collector to separate scheelite from quartz. Micro-flotation experiments demonstrated that DHHAOEP can effectively separate scheelite from quartz within a pH range of 6–9. Artificial mixed ores flotation experiments revealed that at a pH of approximately 8 and a DHHAOEP concentration of 8 × 10^−5^ mol/L, the flotation recovery of scheelite reached 73% with a grade of 54%. The contact angle and Zeta potential measurements showed that the addition of DHHAOEP caused a positive shift in the zeta potential and enhanced the surface hydrophobicity of scheelite. The FTIR, XPS, and DFT analyses further elucidated that DHHAOEP anchored on the scheelite surface through the bonding reaction between its -C(=O)-NHOH moiety and WO_4_^2−^ or Ca active sites on the scheelite surface, forming a five-membered ring. Meanwhile, the existence of the P=O group makes the distance between oxygen atoms in -C(=O)-NHOH very close to that in WO_4_^2+^, which is beneficial to the reaction. The present work aims to develop a novel flotation collector with multi-functional groups to enhance scheelite recovery efficiency and selectivity.

## 1. Introduction

Tungsten, a rare and strategic metal, is indispensable in various industrial applications, including machinery manufacturing, material processing, electronic communications, transportation, and the military industry [1,2,3,4]. On the earth, the main resources of tungsten are derived from scheelite (CaWO_4_) and wolframite ((Mn, Fe)WO_4_) [5,6,7]. As wolframite is continuously consumed, scheelite has gradually become the main source of tungsten resources worldwide [8,9]. However, scheelite commonly coexists with calcite, quartz, and other gangue minerals, which poses significant challenges for the efficient extraction of scheelite. Therefore, the efficient extraction technology of scheelite from gangue has become increasingly important.

Froth flotation, a processing method that involves regulating the difference between the hydrophobicity and hydrophilicity of a mineral surface, is developed as the main method to recover scheelite. In the process of froth flotation, collectors selectively adsorb onto the target mineral particles by means of their polar groups while orienting the non-polar groups towards the water, making the target mineral surface hydrophobic. Subsequently, minerals adsorbed with collectors are more likely to adhere to bubbles, float up with the bubbles, and be enriched, thus achieving flotation separation from other minerals [10]. It is therefore of supreme importance to seek a suitable collector for the successful recovery and separation of scheelite.

Fatty acid collectors are widely used in the flotation separation of scheelite. Kupka et al. [8] reported that fatty acids act as anionic collectors, which adsorb on cationic sites (Ca^2+^) on the surface of scheelite under alkaline conditions. The collector coordinates with each calcium ion through two oxygen atoms in the carboxylate group. However, the selectivity of fatty acid collectors is relatively low, necessitating the addition of other agents such as inhibitors to suppress gangue minerals [11]. Hydroxamic acid collectors containing C(=O)-NHOH groups have excellent selectivity, but their collecting performance still needs to be improved, making them the focus for future development of flotation collectors [12,13]. In order to further improve the flotation performance of hydroxamic acid, metal ions are added to the flotation system of tungsten ore as activators. Zhao et al. [14] found that metal ions Pb^2+^, Ca^2+^, Mn^2+,^ and Fe^2+^ can promote the flotation recovery of scheelite and wolframite by benzyl hydroxamic acid (BHA). BHA has a stronger binding ability to Pb^2+^ than to Ca^2+^, Mn^2+^, and Fe^2+^. Since hydroxamic acid has the advantages of being stable; not easy to hydrolyze, oxidize, and decompose into other substances; and being environmentally friendly, it has great development potential [5]. Therefore, the development of new hydroxamic acid collectors is an important research direction to improve mineral recovery.

In this work, a new collector dihexyl (2-(hydroxyamino)-2-oxoethyl) phosphonate was prepared and used as a flotation collector to separate scheelite from quartz. Micro-flotation, the zeta potential, and the contact angle were conducted to reveal the collecting ability for scheelite and quartz. Furthermore, FTIR, XPS, and DFT calculations were also employed to investigate the adsorption mechanism of DHHAOEP to scheelite.

## 2. Experimental Sections

### 2.1. Materials

**Figure 1:**
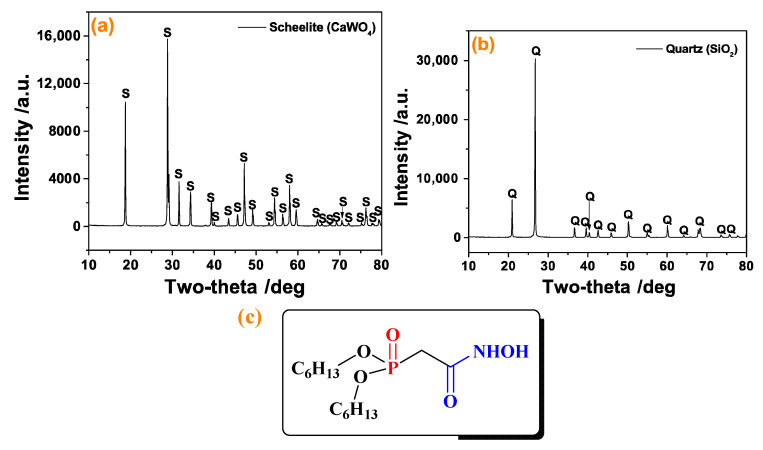
The XRD of scheelite (**a**) and quartz (**b**), and the molecular structure of DHHAOEP (**c**).

The single minerals of scheelite (CaWO_4_) and quartz (SiO_2_) in this experiment were purchased from Guangzhou, Guangdong, China. The X-ray diffraction spectroscopy (XRD) analysis and the X-ray Fluorescence Spectrometry (XRF) was tested on D8 Advance X-ray diffractometer (Bruker, Ettlingen, Germany) and Spectro Midex X-ray fluorescence spectrometer (Spectro, Kleve, Germany), respectively, and the mineral composition of these ore samples is shown in Figure 1 and Table 1. After multiple crushing and sieving, fine granular powders with a particle size of less than 76 μm were obtained. The micro-flotation tests used samples with a particle size between −76 and +38 μm, while the mineral particles less than 5 μm were used for the zeta potential, FTIR, and XPS tests.

The DHHAOEP (shown in Figure 1c) was prepared referring to our previous report [15], and the synthesis and characterization of DHHAOEP were listed in Supporting Information. All the other reagents involved in this experiment were purchased from merchants. Deionized water was used throughout the entire experiment, and the resistivity was 18.2 MΩ·cm^−1^.

### 2.2. Methods

#### 2.2.1. Micro-Flotation Tests

Micro-flotation experiments were completed in a Hallimond tube (see Figure S3); the entire process is divided into four steps: (1) 2 g (scheelite or quartz or a mixture of scheelite and quartz in a mass ratio of 1:1) was weighed in a beaker, then the calculated amount of distilled water was added and stirred using a magnetic stirrer; (2) the slurry pH was regulated to the expected value by adding NaOH or HCl solutions and was stirred for 3 min; (3) an appropriate amount of collector solution was added to make the total volume of the slurry reach 220 mL, then the slurry was stirred for 3 min before standing; and (4) the slurry was then transferred to the Hallimond tube (this process was completed within 3 min at a nitrogen flow rate of 200 mL·min^−1^), and the concentrate and tailings were finally collected. The formulas for calculating the flotation recovery of single minerals or mixed minerals are shown in the Supporting Information. Each condition experiment was repeated three times, and the average of the three results was taken.

#### 2.2.2. Zeta Potential and Contact Angle Tests

Zeta potentials of scheelite and quartz particles were measured using Nano Brook 90 Plus PALS potentiometer (Brookhaven, Farmingville, NY, USA) with 1 × 10^−3^ mol·L^−1^ KCl as the electrolyte [16]. And contact angles were carried out on a JC2000C contact angle analyzer (Zhongchen Digital, Shanghai, China) via the sessile drop method [17]. Detailed operation procedures of the zeta potential and contact angle are shown in the Supporting Information.

#### 2.2.3. FTIR and XPS Tests

The mineral samples before and after DHHAOEP treatment were characterized by Fourier transform infrared spectroscopy (FTIR) and X-ray electron spectroscopy (XPS). The preparation process of the scheelite and quartz used for testing is shown in references [15,17]. Their FTIR spectra were recorded at a 4 cm^−l^ resolution in the 4000–400 cm^−l^ region through KBr disks on a model 740 FTIR spectrometer (Nicolet, Madison, WI, USA). And the XPS measurement was measured on an ESCALAB 250Xi instrument (ThermoFisher, Waltham, MA, USA) with a monochromatic Al Kα radiation (hυ = 1486.71 eV). Thermo Avantage software 5.52 was adopted to analyze the XPS adsorption bands, such as peak position, width, and intensity through Gaussian and Lorentzian functions. The C 1s binding energy set to 284.6 eV was adopted to calibrate the spectrometer.

#### 2.2.4. DFT Calculation

The calculations for DHHAOEP were investigated with the Gaussian 09 software package [18]. Structural optimizations were performed using the B3LYP functional with the 6-31+g (d, p) basis set (empirical dispersion correction GD3(BJ)). The maximum φ (φ_max_) and minimum φ (φ_min_) of these structures were obtained with the help of the Multiwfn code [19].

The Vienna Ab inito Simulation Package (VASP) 5.4.4 [20,21] was used to calculate the adsorption energies of DHHAOEP with scheelite or quartz. The Perdew–Burke–Ernzerhof (PBE) functional within the generalized gradient approximation (GGA) method [22,23] was employed to describe the exchange correlation. The projected augmented wave (PAW) method [24] was captured to calculate the core–valence interactions. Detailed calculation parameters and formulas are shown in the Supporting Information.

## 3. Results and Discussion

### 3.1. DFT Calculation Analyses

The chemical composition of the DHHAOEP solution was analyzed using Advanced Chemical Development (ACD/Labs) software 2023.2.4 [25]. The results are shown in Figure S4a,b. At a pH of about 8, the DHHAOEP molecule loses a hydrogen ion and becomes an anion. In order to explain the flotation mechanism from the perspective of the quantum chemical calculation, the DFT calculation was used to optimize the ionic structure of DHHAOEP (listed in Figure S4c). Some calculated parameters such as MEP (the molecular electrostatic potentials) and HOMO (the highest occupied molecular orbital) are shown in Figure S4d,e.

As can be seen from Figure S4d, the negative charges of DHHAOEP are mainly located on the O atoms of the P=O and C(=O)-NHOH groups, suggesting that the P=O and C(=O)-NHOH groups are the active sites of DHHAOEP. In addition, the negative charge on the O atom of the C(=O)-NHOH group is the largest, about −142.66 kcal/mol, demonstrating that the C(=O)-NHOH group is more reactive than the P=O group. The electron-donating ability of the hydroxamate anion significantly affects its chemical interaction on mineral surface active sites [26].

The electrochemical reactivity between the collector and the mineral was analyzed by HOMO using the frontier molecular orbital theory (FMO) [27]. As displayed in Figure S4e, the HOMO orbital of DHHAOEP anion was mainly centered on the hydroxamate group, and no discernible HOMO orbital was observed on the P=O group. This result also shows that the power supply contribution of the hydroxamic acid group is stronger than that of the phosphonic acid group in the chemical reaction. On the contrary, the phosphonic acid group is in a weak position in the whole process of action.

### 3.2. Micro-Flotation Results

**Figure 2:**
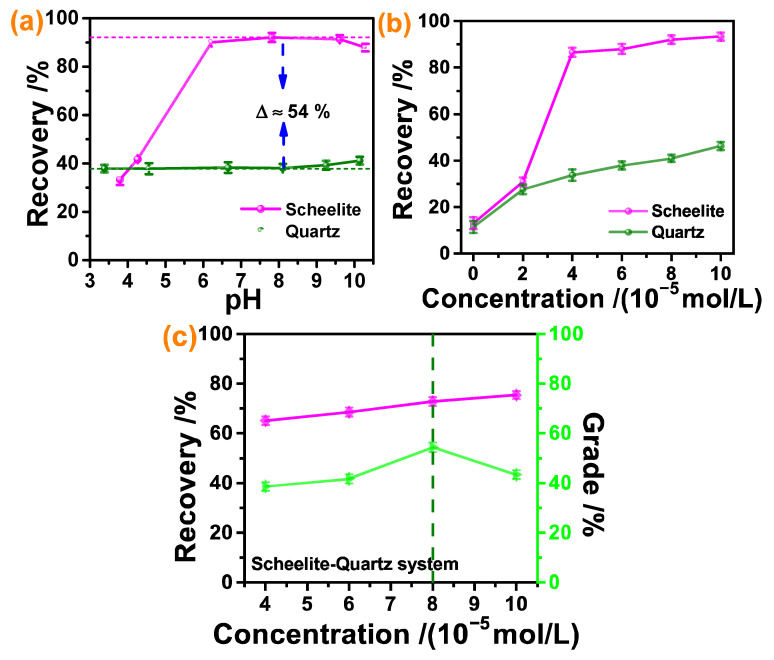
Effects of (**a**) pH (8 × 10^−5^ mol/L DHHAOEP) and (**b**) collector concentration (pH about 8) on the flotation recoveries of scheelite and quartz; (**c**) flotation recoveries and grade of scheelite in artificially mixed minerals at different DHHAOEP dosages (pH about 8).

To understand the potential of DHHAOEP in separating scheelite and quartz, the micro-flotation experiments were conducted with a fixed DHHAOEP concentration of 8 × 10^−5^ mol/L, and the flotation recoveries of scheelite and quartz as a function of pH are displayed in Figure 2a. The results indicated a notable initial increase in the flotation recovery of scheelite, followed by a downward trend as the pH > 9.5, while the flotation recovery of quartz exhibited minimal variation. The maximum difference value in flotation between scheelite and quartz was observed at a pH of 8.0, with a recovery value of 54%. Therefore, pH = 8 was chosen as the most suitable experimental condition in subsequent experiments on scheelite and quartz.

The effects of DHHAOEP concentration on the flotation recovery of scheelite and quartz at pH = 8 were investigated, and the experimental consequences are shown in Figure 2b. It was revealed that the recovery rate of scheelite was significantly better than quartz when DHHAOEP concentration exceeded 4 × 10^−5^ mol/L. Moreover, 8 × 10^−5^ mol/L of DHHAOEP was enough to enhance the flotation recovery of scheelite to 92%, whereas that of quartz was only 38%.

To demonstrate that DHHAOEP has the power to separate scheelite from quartz, an artificial mixed mineral flotation test was conducted. As shown in Figure 2c, at pH ~8, the recoveries of scheelite continued to increase with the increasing of DHHAOEP concentration, while the grade first increased and then decreased. As the DHHAOEP concentration increased from 4 × 10^−5^ mol/L to 8 × 10^−5^ mol/L, the flotation recovery of scheelite increased from 65% to 73%, and the mineral grade increased from 39% to 54%. The concentration of DHHAOEP continued to increase to 1 × 10^−4^ mol/L; the recovery of scheelite increased slightly, and the grade decreased significantly. The reason why the grade decreases with the increase in DHHAOEP concentration may be that the increase in DHHAOEP promotes the flotation recovery of quartz, resulting in a decrease in the grade of scheelite in the concentrate. Based on the above analysis, DHHAOEP had an excellent separation impact on scheelite and quartz and could be recognized as a resultful collector for separation and purification scheelite associated with quartz.

### 3.3. Zeta Potential Analysis

**Figure 3:**
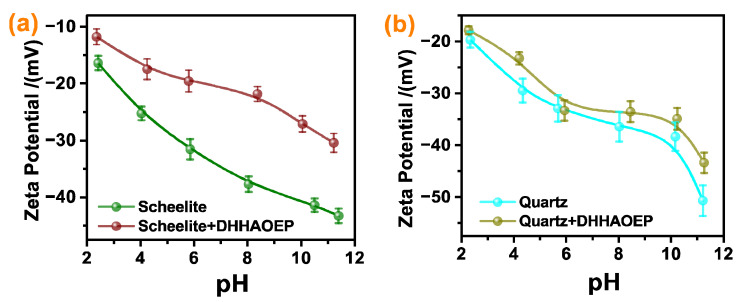
Zeta potential of scheelite (**a**) and quartz (**b**) before and after treatment with 1 × 10^−4^ mol/L DHHAOEP.

The effect of pH on the zeta potential of scheelite and quartz particles before and after DHHAOEP treatment was tested, with the experimental data displayed in Figure 3. It indicated that the zeta potential values of scheelite and quartz particles were observed to be negative across the pH range of 2–12; this was consistent with the previous literature [28]. When DHAHOEP was added, the zeta potential of scheelite shifted significantly to a positive value, inferring that DHAHOEP can be anchored to the surface of scheelite. However, after the adsorption of DHHAOEP, the zeta potential of quartz only shifted slightly positively, announcing that DHHAOEP has an extremely limited ability to act on the quartz surface. At pH 8, the zeta potential of scheelite exhibited a positive shift of 15.9 mV (from −37.7 mV to −21.8 mV) with the addition of 1 × 10^−4^ mol/L DHHAOEP, whereas quartz only experienced a marginal shift of 2.9 mV (from −36.4 mV to −33.5 mV). These findings indicated that the adsorption performance of DHHAOEP on the surface of scheelite was superior to quartz. Therefore, DHHAOEP has selectivity for scheelite and quartz to some extent.

### 3.4. Contact Angle Analysis

**Figure 4:**
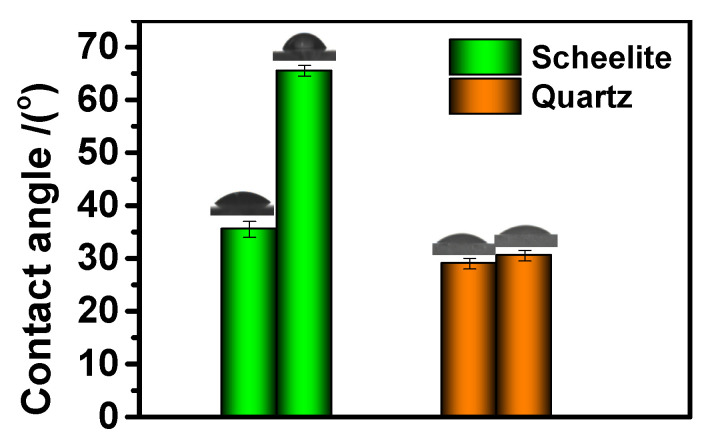
Contact angle of scheelite and quartz before and after being immersed in 5 × 10^−4^ mol/L DHHAOEP solution for 30 min.

The contact angle values of water droplets on scheelite and quartz surfaces before and after treatment with DHHAOEP were measured at 25 °C and the consequences listed in Figure 4. Before DHHAOEP treatment, the water drop contact angles of clean scheelite and quartz were 35.5 and 29.00°, independently; after adsorption of 5 × 10^−4^ mol/L DHHAOEP solution at pH ~8 for 30 min, the water drop contact angles of scheelite and quartz increased to 65.5 and 30.50, independently. The results showed that the presence of DHHAOEP significantly improved the surface hydrophobicity of scheelite but had little effect on quartz. This difference in hydrophobicity contributes to the distinct flotation behavior observed between scheelite and quartz when treated with DHHAOEP, as illustrated in Figure 2.

### 3.5. FTIR Analysis

To better understand the interaction of DHHAOEP with scheelite and quartz and their separation mechanism, we performed FTIR analysis on scheelite and quartz before and after DHHAOEP interaction.

**Figure 5:**
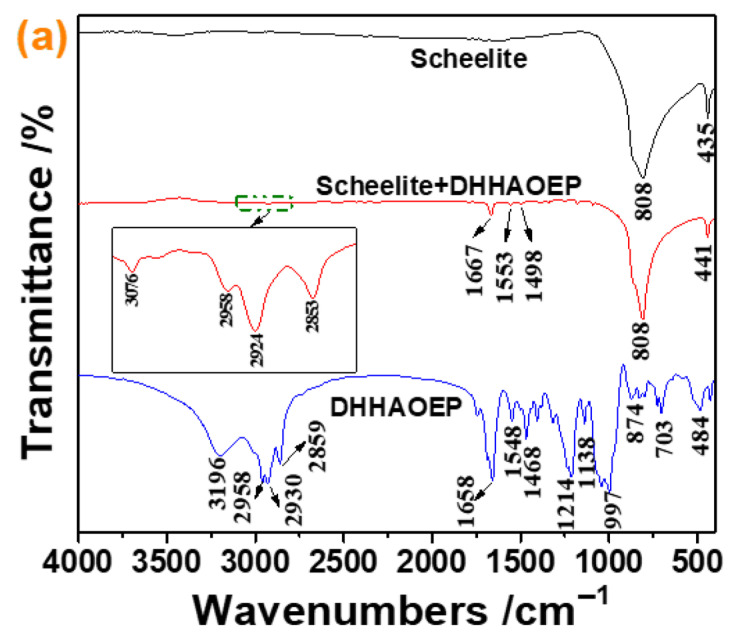

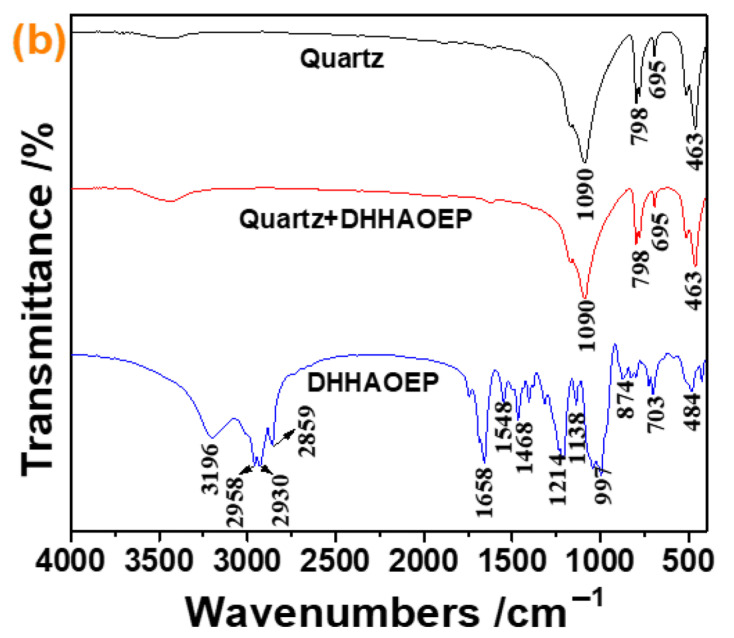
FTIR spectra of DHHAOEP, scheelite, and DHHAOEP-treated scheelite (**a**) and DHHAOEP, quartz, and DHHAOEP-treated quartz (**b**).

Figure 5a shows the results of the spectrum for DHHAOEP. It indicated that the O-H and N-H stretching vibrations of the -C(=O)-NHOH group appeared at 3196 cm^−1^ [29,30,31,32]. The peaks at ~2958, 2930, and 2859 cm^−1^ were attributed to the C-H stretching bands of the -CH_2_- and -CH_3_ groups [30,33]. The peaks at 1658 and 1548 cm^−1^ were due to the C=O and -C-N- stretching vibrations of the -C(=O)-NH- group [30], respectively. The adsorption peaks at 1214 and 997 cm^−1^ were assigned to the P=O and P-O-C stretching bands of the -P(=O)-O-C group [34,35,36,37], respectively. After treatment with DHHAOEP, the C-H stretching vibrations of the -CH_2_- and -CH_3_ groups at 2958, 2924, and 2853 cm^−1^ appeared on the scheelite surfaces, and the peaks at 1658 and 1548 cm^−1^ were shifted to 1667 and 1553 cm^−1^, respectively. These findings demonstrated the strong chemical adsorption of DHHAOEP on the scheelite surface.

Figure 5b shows the FTIR spectra of quartz before and after DHHAOEP treatment. The results showed that the FTIR spectra of quartz remained unchanged after DHHAOEP treatment, indicating that there was no special chemical reaction between DHHAOEP and quartz. This phenomenon is consistent with the experimental research results of Zeta potential.

### 3.6. XPS Analysis

The XPS spectra of DHHAOEP, scheelite, and scheelite treated with DHHAOEP were shown in Figure S5, and the atomic concentrations were shown in Table 2. Table 2 showed that the atomic concentration ratio of O and P to N was 5.27:1.15:1, which closely aligns with the theoretical value of 5:1:1 for the DHHAOEP molecule. After the DHHAOEP treatment, the peaks of N and P elements appeared on the scheelite surface, and the atomic concentrations of O, W, and Ca decreased, which may have been caused by the adsorption of DHHAOEP on the scheelite surface. Since DHHAOEP was adsorbed and covered on the surface of scheelite, the detected element content of scheelite decreased to a certain extent.

**Figure 6:**
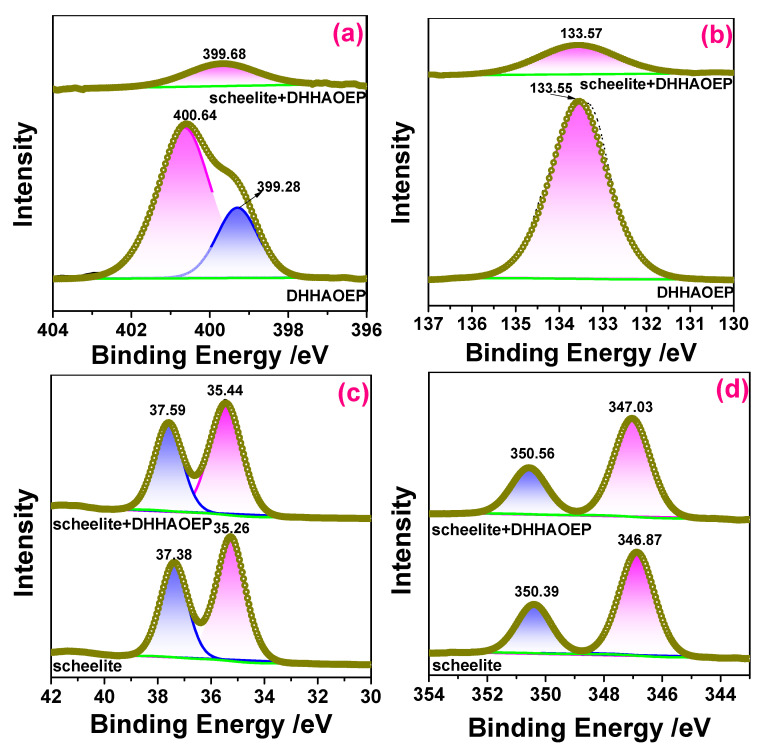
High-resolution XPS of N 1s (**a**), P 2p (**b**), W 4f (**c**), and Ca 2p (**d**).

The high-resolution XPS spectra of DHHAOEP, scheelite, and DHHAOEP-treated scheelite are analyzed and displayed in Figure 6; the parameters and attributions of various elements N, P, W, and Ca are shown in Tables S1–S4.

As shown in Figure 6a and Table S1, the N 1 s XPS band of DHHAOEP is divided into two peaks near 399.28 and 400.64 eV, which are attributed to the two groups -C(=O)-NHO- and -C(=O)-NHOH, respectively [38]. The possible reason is that the acidification step in the synthesis of hydroxamic acid is not complete, resulting in some hydroxamate not being converted into hydroxamic acid. After DHHAOEP treatment, a single N1s peak appeared on the surface of scheelite at 399.68 eV, indicating that the N element in the DHHAOEP molecule [9] may be a part of the adsorption process between DHHAOEP and scheelite.

Figure 6b and Table S2 show that DHHAOEP exhibits a P 2p XPS band at 133.55 eV, which belongs to the P atom of the P=O group [39]. For DHHAOEP-treated scheelite, P 2p XPS appeared at around 133.57 eV, suggesting the P=O bond remained unchanged during the reaction of DHHAOEP molecules with scheelite. This is similar to the DFT analysis results.

Figure 6c,d and Tables S3 and S4 present the W 4f or Ca 2p XPS of scheelite before and after DHHAOEP condition. The W 4f XPS bands of the pristine scheelite consisted of two peaks at 35.36 eV and 37.38 eV, and the Ca 2p XPS peaks of scheelite occurred at 346.87 eV and 350.39 eV [40,41]. After the DHHAOEP condition, the W 4f XPS bands exhibited a shift towards lower values at 35.44 eV and 37.59 eV, while the Ca 2p XPS peaks displayed a shift towards higher values at 347.03 eV and 350.56 eV. These findings indicate that the presence of DHHAOEP on the scheelite surface induces a chemical modification in the W and Ca atoms, indicating potential chemisorption of DHHAOEP onto scheelite surfaces.

### 3.7. Discussion

In order to further explore the adsorption mechanism of DHHAOEP anions on the surfaces of scheelite and quartz from the perspective of microscopic molecular dynamics, the surface interaction energy of scheelite and quartz after the adsorption of DHHAOEP was calculated by DFT. The results are displayed in Figure S4 and Table 2.

**Figure 7:**
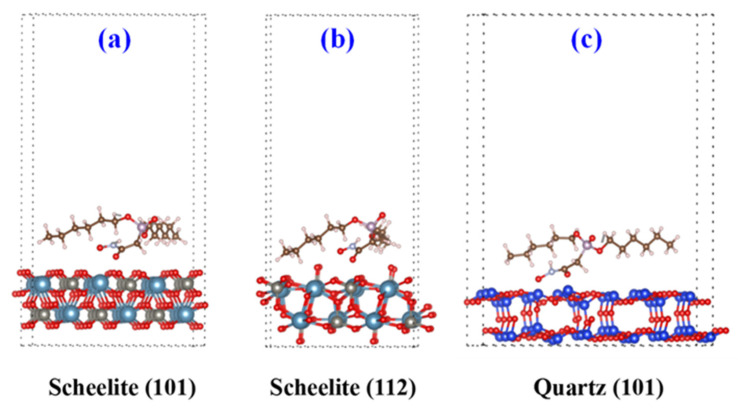
Molecular models of scheelite and quartz surfaces: (**a**) scheelite (101) + DHHAOEP^−^, (**b**) scheelite (112) + DHHAOEP^−^, and (**c**) quartz (101) + DHHAOEP^−^.

Figure 7 illustrated that whether on the scheelite (112) or (101) faces, or on the quartz (101) face, the hydroxamate anions in the DHHAOEP are positioned closer to the mineral surface than the P=O groups, suggesting higher chemical reactivity of the hydroxamate anions compared to that of the P=O groups, and DHHAOEP bonded the active sites on the mineral surface through the hydroxamate anions. The results in Table 3 show that the adsorption energy of DHHAOEP on scheelite is significantly higher than that on quartz, indicating that DHHAOEP has a stronger binding ability with scheelite and the formed surface material is more stable, which provides strong evidence for the flotation of scheelite from quartz in this experiment.

**Figure 8:**
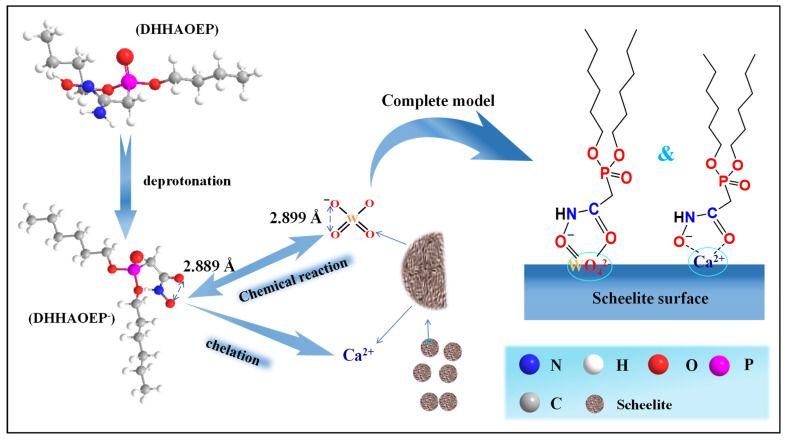
The potential adsorption mechanism for scheelite with DHHAOEP^−^.

As per the previous reports [28,42], scheelite has a tetragonal crystal structure, and the crystal parameters are a = b = 5.243 Å, c = 11.376 Å, and α = β = γ = 90°. The (1 1 1) and (1 1 2) surfaces of scheelite have lower surface energy. These two cleavage planes are usually produced during the crushing and fine grinding of scheelite. Quartz has a hexagonal crystal structure with a = 4.913 Å and c = 5.405 Å [43,44]. The cleavage of quartz usually occurs along the (101) surface, and its surface energy value is the lowest among the three. Based on the above lattice parameters, the distance between the two adjacent oxygen atoms in WO_4_^2−^ of scheelite is 2.899 Å, and that in SiO_2_ of quartz is 3.142 Å. Meanwhile, through DFT calculation, the distance between the two oxygen atoms in the hydroxamate anion of the DHHAOEP is 2.889 Å, which is approximately equal to the distance between the two adjacent oxygen atoms in WO_4_^2−^ but much smaller than that in SiO_2_, indicating that C(=O)-NHO^−^ can form a five-membered ring 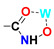 configuration with W in WO_4_^2−^, as shown in Figure 8. The results are in agreement with those calculated by FTIR, XPS, and DFT. At the same time, the binding form of such hydroxamic acid with minerals is similar to the N-((hydroxyamino)-alkyl) alkyl amide surfactants (NHOD^−^) studied by Deng [28]. However, the bond length between two adjacent oxygen atoms in NHOD^−^ is 2.842 Å, while that in DHHAOEP^−^ is 2.889 Å, which is closer to the 2.899 Å between two adjacent oxygen atoms in scheelite. Therefore, it can be inferred that the presence of the P=O group in DHHAOEP increases the distance between adjacent oxygen atoms in C(=O)-NHO^−^, which can better react with the active sites on the surface of scheelite.

## 4. Conclusions

In this work, a novel collector DHHAOEP containing both C(=O)-NHOH and P=O groups was designed and synthesized. Through flotation tests; zeta potential; contact angle; and FTIR, XPS, and DFT calculations, the flotation performance of scheelite and quartz was discussed, and the adsorption mechanism of DHHAOEP and scheelite was revealed. According to the existing experimental data, the following analysis conclusions were obtained.

The flotation results demonstrated that DHHAOEP had excellent harvesting ability for scheelite. At pH 6~9, DHHAOEP had an excellent separation impact on scheelite and quartz. The results of zeta potential and contact angle demonstrated that DHHAOEP bonded on the scheelite surface and evolved the hydrophobicity of the scheelite surface.

FTIR and XPS spectra revealed that DHHAOEP interacts with scheelite only through the hydroxamate group in its molecular structure, and this interaction is mainly achieved through chemical adsorption. Furthermore, the DFT calculation results also indicate that DHHAOEP has a stronger binding ability with scheelite. This is also the fundamental reason why DHHAOEP can float and separate scheelite from quartz. Although the addition of phosphoryl groups does not directly participate in chemical adsorption, it promotes the optimization of hydroxamic acid groups in DHHAOEP, thereby facilitating the flotation performance and selectivity of scheelite.

## Figures and Tables

**Table 1:** Chemical composition of scheelite and quartz sample.

Sample	WO_3_	Al_2_O_3_	CaO	Au	SiO_2_
Scheelite	77.12	0.780	0.107	0.306	1.11
Quartz	0.0146	0.923	<0.042	<0.02	98.54

**Table 2:** Atomic concentration of elements for DHHAOEP, scheelite, and DHHAOEP-modified scheelite as determined by XPS.

Substance	Atomic Concentration/%					
	C 1s	N 1s	O 1s	P 2p	W 4f	Ca 2p
DHHAOEP	66.99	4.45	23.44	5.12	-	-
Scheelite	25.29	-	51.05	-	11.43	12.24
DHHAOEP-modified Scheelite	25.59	0.91	49.14	1.66	10.92	11.78

**Table 3:** Interaction energies of scheelite and quartz surfaces with DHHAOEP.

Reaction Conditions	*E*_Mineral_ (kJ/mol)	*E*_Reagents_ (kJ/mol)	*E*_Total_ (kJ/mol)	Δ*E* (kJ/mol)
Scheelite (101) + DHHAOEP	−86,003.52	−26,933.76	−113,214.72	−277.44
Scheelite (112) + DHHAOEP	−76,201.92	−26,933.76	−103,390.08	−254.40
Quartz (101) + DHHAOEP	−99,743.04	−26,933.76	−126,876.48	−199.68

## Data Availability

The original contributions presented in this study are included in the article/Supplementary Material. Further inquiries can be directed to the corresponding authors.

## Supplementary Materials

The following supporting information can be downloaded at: https://www.mdpi.com/article/10.3390/molecules30173607/s1. Synthesis, characterization, and DFT calculations of DHHOEP, and the survey XPS spectrum and detailed analysis of N, P, W, and Ca elements.
