# Supplementary material for: Dihexyl (2-(Hydroxyamino)-2-Oxoethyl) Phosphonate as a Novel Collector for Flotation Separation of Scheelite and Quartz
Source: Molecules. 2025 Sep 3;30(17):3607. doi: 10.3390/molecules30173607 (PMC12430788; doi:10.3390/molecules30173607)
Supplement: Supplementary file 1 [file molecules-30-03607-s001.zip › molecules-3786206-supplementary.pdf]

Supporting Information

for

Dihexyl (2-(Hydroxyamino)-2-oxoethyl) Phosphonate as a Novel Collector for Flotation Separation of Scheelite and Quartz

Jingjing Xiao [1], Pan Xiao [2], Yongjun Miao [1], Sisi Liu [1], Jia Tu [1], Qing Tang [3], Changzhu Li [1,*], Zhihong Xiao [1,*] and Rukuan Liu [1]

[1] State Key Laboratory of Woody Oil Resources Utilization, Hunan Academy of Forestry, Changsha 410004, China; xjj0806@hnlky.cn (J.X.); yjmiao1971@126.com (Y.M.); liusisi274@126.com (S.L.); liurukuan@gmail.com (R.L.)

[2] The College of Resources and the Environment, Central South University of Forestry and Technology, Changsha 410004, China; 19146808384@163.com

[3] Center for Industrial Analysis and Testing, Guangdong Academy of Sciences, Guangzhou 510645, China; tangqing822@163.com

* Correspondence: lichangzhu2013@aliyun.com (C.L.); xiaozhihong@hnlky.cn (Z.X.)

S 2.1 Materials

The conveniently prepared routine of DHHAOEP was displayed in Scheme S1. Methyl chloroacetate (10.96g, 0.10 mol) was put into a 250-mL three-neck round bottom flask. Trihexyl phosphite (35.16 g, 0.10 mol) was added under stirring. The mixture reacted at 160 °C for 3 h. The intermediate methyl 2-(bis(hexyloxy)phosphoryl) acetate (**2**, 31.89 g) was collected as a colorless transparent liquid with a 98.97 % yield.

To 7.72 g (0.11 mol) hydroxylamine hydrochloride, 50 mL methanol were introduced. Then, 8.00 g (0.20 mol) sodium hydroxide were added in batches. When the reaction between hydroxylamine hydrochloride and sodium hydroxide was finished, methyl 2-(bis(hexyloxy)phosphoryl) acetate was added dropwise to the mixture. After stirring for 2 h at 20 °C, 3 M HCl was employed to acidize the mixture to pH ~6.00. After filtering, DHHAOEP (**3**, 30.66 g) was obtained by vacuum distillation at 0.06 MPa

and 60 °C with a 94.92 % yield.

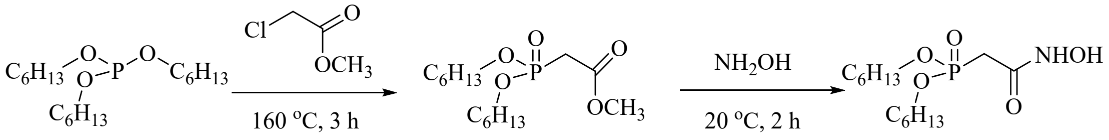

1 2 3

Scheme S1 Synthesis route of DHHAOEP

Characterization of DHHAOEP: White flaky crystals. ^{1}H NMR (DMSO-*d*6, 400 MHz, see Figure S1(a)), δ_H: 10.45 (s, 1H, -OH), 8.95 (s, 1H, -NH), 3.93-3.98 (t, 4H, $-CH_2-$), 3.62 (s, 1H, $-CH_2-$), 2.68 (s, 1H, $-CH_2-$), 1.54-1.61 (t, 4H, $-CH_2-$), 1.27-1.33 (t, 12H, $-CH_2-$), 0.85-0.88 (t, 6H, $-CH_3$); ^{13}C NMR (DMSO-*d*6, 100 MHz, see Figure S1(b)), δ_C: 161.64 (C=O), 66.15 ($-CH_2-O$), 39.89 ($-CH_2-$), 39.47 ($-CH_2-$), 39.06 ($-CH_2-$), 31.20 ($-CH_2-$), 22.45 ($-CH_2-$), 14.28 ($-CH_3$); ^{31}P NMR (DMSO-*d*6, 162 MHz, see Figure S1(c)), δ_p: 22.60 (P=O). HR ESIMS calculated for [C14H30NO5P]+= 324.2100; found=324.2086, see Figure S2.

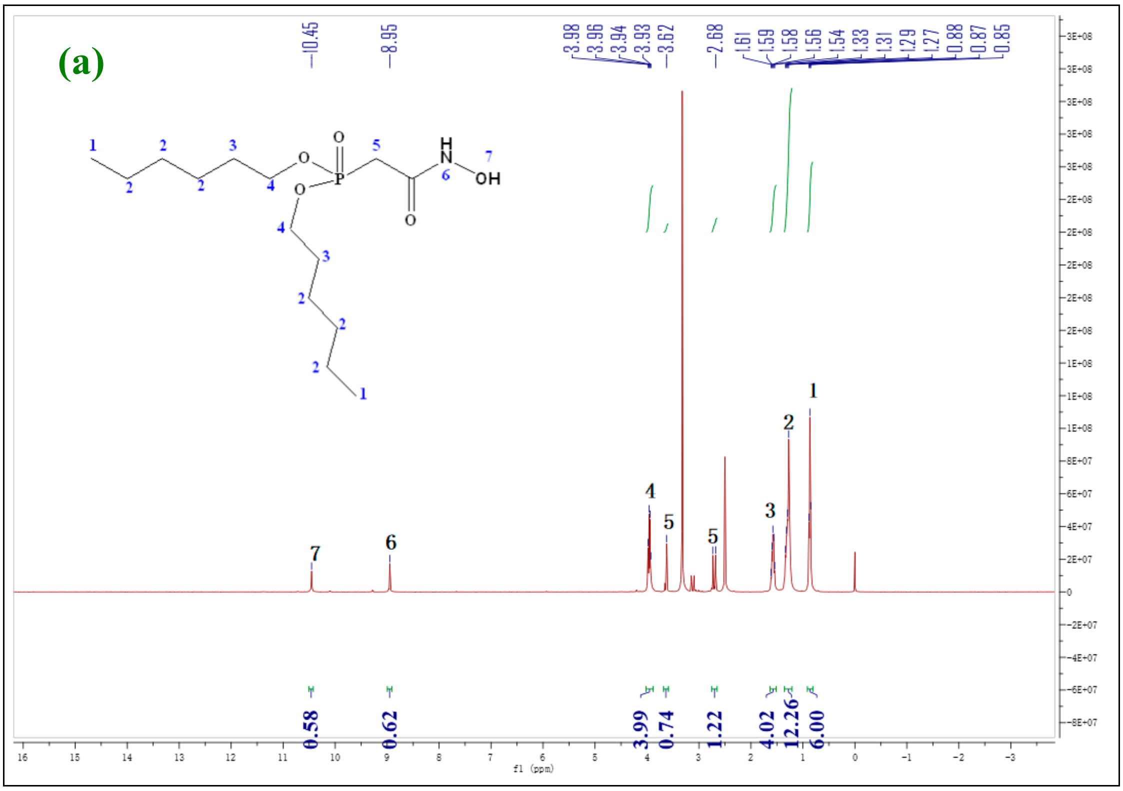

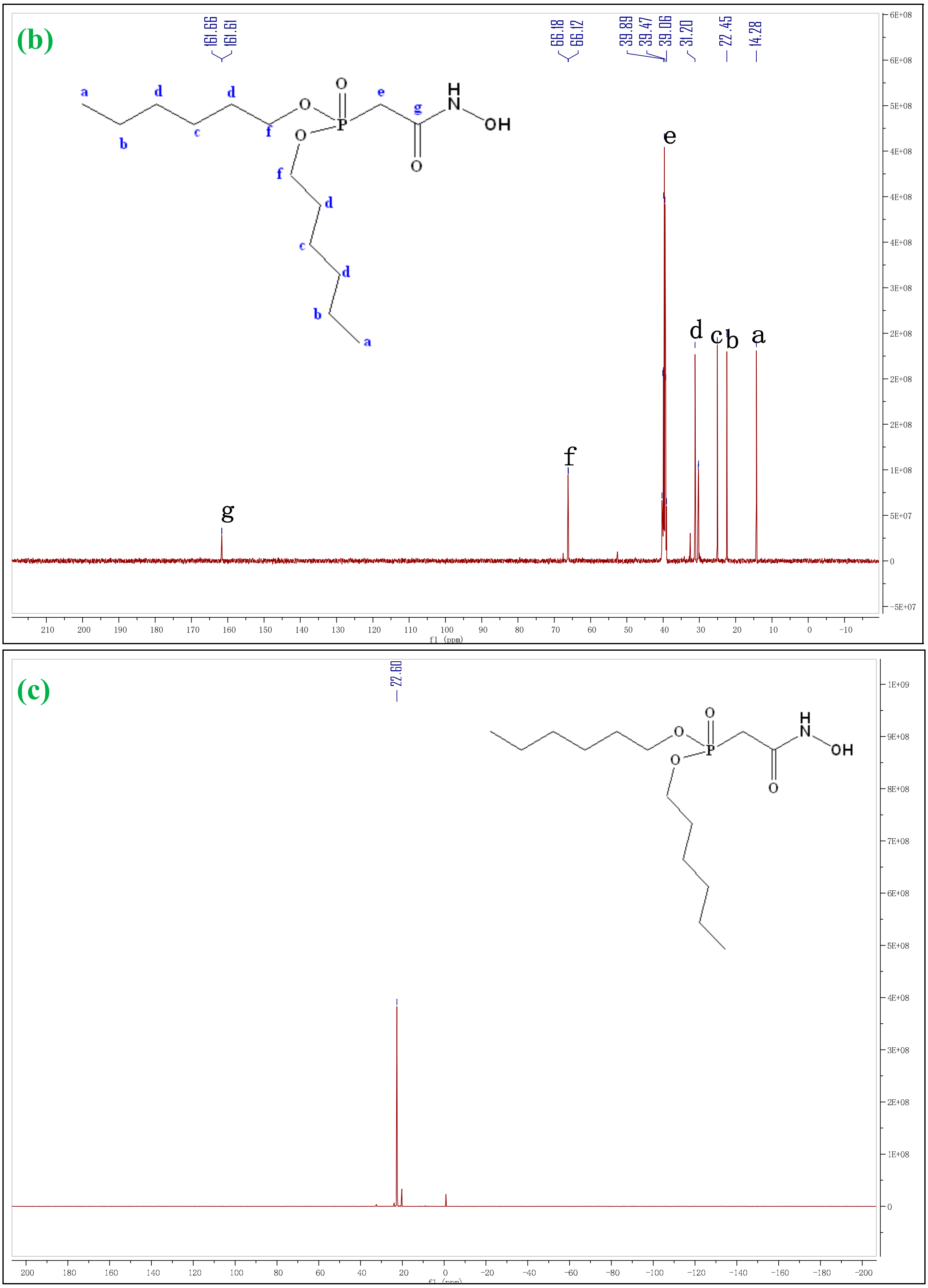

Figure S1 ^{1}H NMR (a), ^{13}C NMR (b) and ^{31}P NMR (c) of DHHAOEP

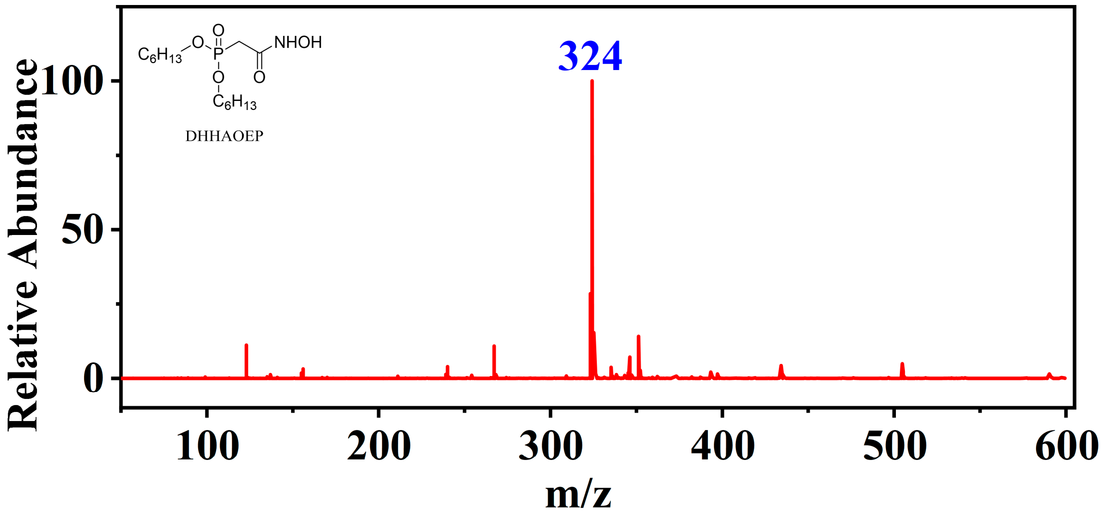

Figure S2 The HRMS spectrum of DHHAOEP extractant

S 2.2.1 Micro-flotation tests

The concentrates and tailings after the flotation of the above-mentioned scheelite and quartz were weighed, and the recovery were calculated using the following three formulas. Formula (1) is a method for calculating the flotation recovery of a single mineral, and formulas (2) and (3) are methods for calculating the recovery of scheelite and quartz in mixed ores, respectively.

$$\omega = \frac{m_1}{m_1 + m_2} \times 100\% \tag{1}$$

$$\omega_{scheelite} = \frac{m_1 \times \varphi_1}{m_1 \times \varphi_1 + m_2 \times \varphi_2} \times 100\% \tag{2}$$

$$\omega_{quartz} = \frac{m_1 \times (1 - \varphi_1)}{m_1 \times (1 - \varphi_1) + m_2 \times (1 - \varphi_2)} \times 100\% \tag{3}$$

Where m_1 and m_2 represent the mass of concentrate and tailings respectively; φ_1 and φ_2 represent the grade of scheelite in concentrate and tailings, respectively; ω represents the flotation recovery.

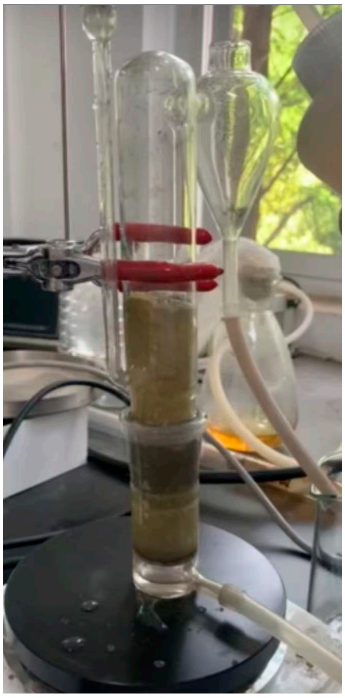

Figure S3 Experimental apparatus of micro-flotation tests

S 2.2.2 Zeta potential and contact angle tests

First, 50 mg of mineral samples (<5 μm) were placed in the electrolyte solution with or without DHHAOEP. Then, the pH of the solution was adjusted to the predetermined value with HCl and KOH solutions, and the measurement was performed after stirring for 5 min. The zeta potential values reported in this study were the average of three independent measurements with a maximum standard deviation of ±2.5 mV.

Prior to each measurement, the mineral mounted in resin was freshly polished and then rinsed with deionized water and blew dry with nitrogen. Subsequently, the mineral was soaked in the DHHAOEP solution with adjusted concentration and pH for a period of time. Then took out the mineral sample and washed it repeatedly with distilled water for at least 5 times to ensure that the collector residue on the mineral surface was removed as much as possible. Finally, dried the mineral surface with pure N2, and then measured the static contact angle of each. The average of three independent measurements was taken as the experimental result.

S 2.2.6 DFT calculation

The energy cutoff for plane wave expansions was set to 480 eV, and the 3×3×1 Monkhorst-Pack grid k-points were selected to sample the Brillouin zone integration. The vacuum space is adopted 15 Å above the surfaces to avoid periodic interactions. The structural optimization was completed for energy and force convergence set at 1.0×10^{-4} eV and 0.02 eV $Å^{-1}$, respectively.

The adsorption energy can be calculated according to the following formula S (1):

$$E_{ads} = E_{(mineral+DHHAOEP)} - E_{mineral} - E_{DHHAOEP} \quad (1)$$

where E_{ads} represents the adsorption energy, $E_{(mineral+DHHAOEP)}$ is the calculated energy of adsorption configuration, $E_{(mineral)}$ and $E_{(DHHAOEP)}$ mean the calculated energy of mineral and collector, respectively.

S3.1 DFT calculation analyses

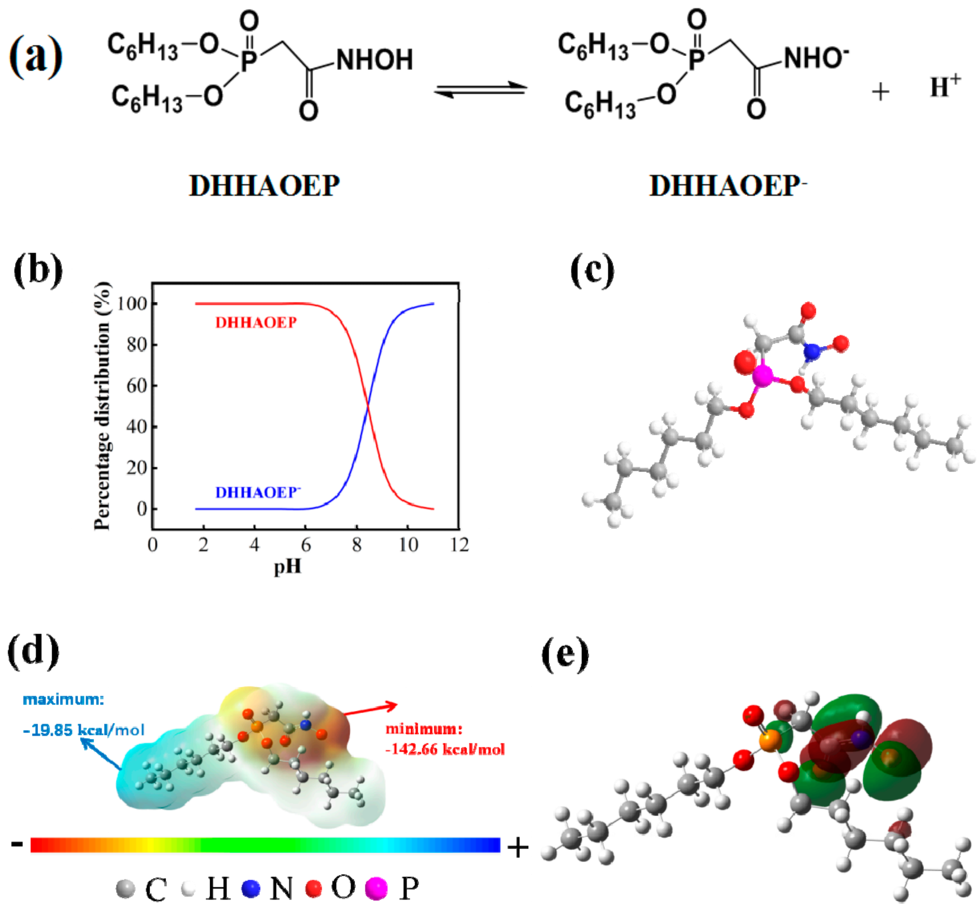

Figure S4 Ionization of DHHAOEP (a) and distribution percentage of various dissociated species (b), the optimized ionic structure of DHHAOEP (c), MEP (d), HOMO (e).

S3.6 XPS analysis

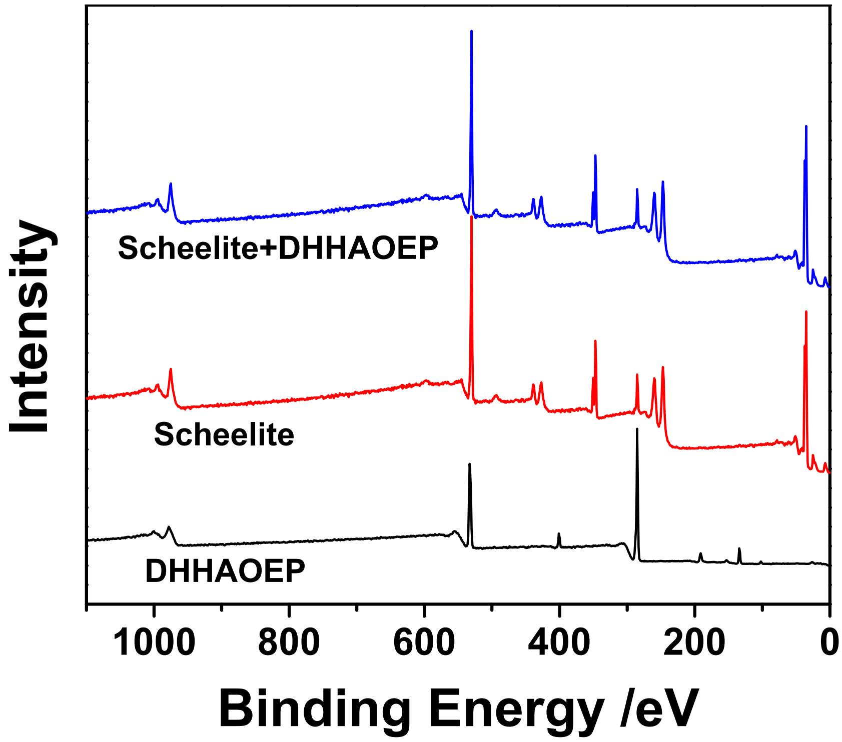

Figure S5 The survey scan XPS of DHHAOEP, scheelite and DHHAOEP-treated scheelite

Table S1 The results of high-resolution N1s XPS

	DHHAOEP		DHHAOEP treated Scheelite
Binding Energy/eV	399.28	400.64	399.68
FWHM[a]/eV	1.28	1.67	2.18
Percentage/%	25.93	74.07	100
Assignment	C(=O)-NHO$^-$	C(=O)-NHOH	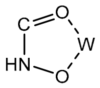

Table S2 The results of high-resolution P2p XPS

	DHHAOEP	DHHAOEP treated Scheelite
Binding Energy/eV	133.55	133.57
FWHM[a]/eV	1.59	2.20
Percentage/%	100	100
Assignment	P=O	P=O

Table S3 The results of high-resolution W4f XPS

	Scheelite		DHHAOEP treated Scheelite	
Binding Energy/eV	35.26	37.38	35.44	37.59
FWHM[a]/eV	1.36	1.25	1.51	1.30

Percentage/%	59.17	40.83	59.52	40.48
Assignment	W4f 7/2	W4f 5/2	W4f 7/2	W4f 5/2

Table S4 The results of high-resolution Ca2p XPS

	Scheelite		DHHAOEP treated Scheelite	
Binding Energy/eV	346.87	350.39	347.03	350.56
FWHM[a]/eV	1.49	1.45	1.54	1.50
Percentage/%	68.49	31.51	68.49	31.51
Assignment	Ca 2p 3/2	Ca 2p 1/2	Ca 2p 3/2	Ca 2p 1/2

FWHM[a]: full width of half maximum
